# Prevalence and comorbidity of mental disorders among adolescents living in residential youth care

**DOI:** 10.1007/s00787-015-0700-x

**Published:** 2015-03-07

**Authors:** Thomas Jozefiak, Nanna Sønnichsen Kayed, Tormod Rimehaug, Anne Kristine Wormdal, Ann Mari Brubakk, Lars Wichstrøm

**Affiliations:** 1 Faculty of Medicine, Regional Center for Child and Youth Mental Health and Child Welfare, Medical Technical Research Centre, Norwegian University of Science and Technology, Postbox 8905, 7491 Trondheim, Norway; 2 Department of Child and Adolescent Psychiatry, St. Olav’s University Hospital, Trondheim, Norway; 3 Department of Laboratory Medicine, Children’s and Women’s Health, Norwegian University of Science and Technology, Trondheim, Norway; 4 Department of Pediatrics, St. Olav’s University Hospital, Trondheim, Norway; 5 Department of Psychology, Norwegian University of Science and Technology, Trondheim, Norway

**Keywords:** Residential youth care, Adolescents, Prevalence, Comorbidity, Mental disorders, CAPA

## Abstract

Most adolescents are placed in residential youth care (RYC) because of severe psychosocial strains and child maltreatment, which represent risk factors for developing mental disorders. To plan RYC units and ensure that residents receive evidence-based psychiatric interventions, it is necessary to obtain reliable and valid prevalence estimates of mental disorders in this population. However, there is a lacuna of research on diagnoses derived from standardized clinical interviews. The aim of this study was to assess the prevalence and comorbidity of mental disorders applying diagnostic interviews in an entire population of adolescents living in RYC in Norway. All young people in RYC were invited to participate in the study. Eighty-six RYC institutions with 601 eligible adolescents were included and 400 adolescents, 12–20 years old, participated in the study, yielding a response rate of 67 %. Anonymous Child Behaviour Checklist scores for 141 (70 %) of the declining residents were also available, allowing diagnoses according to the Diagnostic and Statistical Manual of Mental Disorders Fourth Edition (DSM-IV) for 541 youths to be estimated. Diagnoses were assessed by trained interviewers with the Child and Adolescent Psychiatric Assessment interview (CAPA). Seventy-six point two per cent (71.5–80.8 CI 95 %) of adolescents received at least one 3-month DSM-IV diagnosis. Prevalence rates for internalizing psychiatric disorders were higher than for behavioural disorders. Comorbidity was high between these two groups. Mental disorders were prevalent among children and youth in RYC. Our results create major concerns and challenge the existing organization of the RYC system.

## Introduction

Most adolescents are placed in residential youth care (RYC) because of severe psychosocial strains and child maltreatment, which are well known as risk factors for developing mental disorder [1]. In Norway, the official policy is that foster care is the preferred form of placement and RYC institutions are a last resort [2]. To plan RYC units and provide evidence-based and individually tailored psychiatric interventions for adolescents living in RYC, it is necessary that knowledge based on reliable and valid prevalence estimates of mental disorders and their comorbidity is available. Effective, evidence-based psychiatric treatment approaches and interventions are available for most psychiatric disorders in adolescents. However, to adequately provide such interventions to adolescents in RYC units, it is necessary to have knowledge about the residents’ psychiatric diagnoses and comorbidity with other diagnoses. Furthermore, design of the scope and types of RYC institutions should be based on such knowledge. Finally, providing adequate help for adolescents with mental disorders who are living in RYC units is also important in a social–economic cost–benefit perspective, because adolescents in the child welfare system evidently have major difficulties in school functioning and completing education [3–5] and are at higher risk for substance abuse and criminal behaviour [6–9], thereby generating social costs [2, 10].

Higher rates of emotional and behavioural problems among youth in the child welfare system seem well substantiated by a range of studies using questionnaire-type measurements and rating scales [11–13]. However, defining psychiatric disorder solely in terms of psychiatric symptoms can result in implausibly high caseness rates [14]. The use of rating scales to estimate diagnostic prevalence may result in high rate of false positives [15]. This may be the case because the decision regarding whether a symptom is present or not is left to the subject: one adolescent might interpret ‘often’ differently to another adolescent—how often is ‘often’? Diagnosis, be it based on the Diagnostic and Statistical Manual of Mental Disorders (DSM) [16] or the International Classification of Mental and Behavioural Disorders (ICD) [17], requires not only the presence of symptoms, but also the fulfilment of specified onset, duration and functional impairment criteria. Such additional criteria are not usually included in rating scales. Therefore, to obtain valid prevalence figures based on diagnostic classification, the assessment should preferably be conducted by a trained professional using a standardized psychiatric interview.

Unfortunately, there is a lacuna of research applying diagnostic criteria [18]. There appear to be only five studies that have used structured diagnostic interviews in RYC units to yield DSM-IV or ICD-10 diagnoses [18–22]. There are some additional studies that used indirect methods, i.e. one study applied a systematic protocol for staff assessing DSM-IV diagnoses based on the investigational proceedings normally followed at the institutions [23], whereas another inquiry [24] used the Development and Well-Being Assessment (DAWBA) [14] and reported ICD-10 diagnoses [17]. Using the DAWBA implies that nonclinical interviewers administer a structured interview to parents and adolescents aged 11–16 years about psychiatric symptoms and resultant impact. “Interviewers use open-ended questions and supplementary prompts to get informants to describe the problems in their own words. Computer-generated summary sheets and diagnoses form a convenient starting point for experienced clinical raters, who decide whether to accept or overturn the computer diagnosis (or lack of diagnosis) in the light of their review of all the data, including transcripts” [14]. We expect a higher prevalence of mental disorders in RYC units than in other areas of the child welfare system, e.g. foster care. In Norway, foster care is the preferred form of placement and RYC institutions are a last resort [2]. Also, juvenile detention centres do not exist in Norway, so children under the age of 16 with criminal behaviour can be placed in RYC institutions. Youths in RYC institutions can therefore be presumed to be a high-risk population with drug and conduct-related problems, which are associated with high rates of mental health disorders. To avoid bias, in our review of knowledge base chronicled below, we therefore include research on RYC units only, excluding studies using structural diagnostic interviews on other child populations looked after by local authorities, such as children and adolescents in foster care, if they did not also include a subsample of adolescents living in RYC.

Of the seven studies mentioned above, the early McCann et al. study from Great Britain reported from a sample of 88 adolescents 13–17 years old, who were looked after by a local authority. A minority (we could not identify the exact number) were living in residential units, the majority in foster care. Forty-seven were high scorers on the Child Behaviour Checklist (CBCL) [25], 10 refused or were missing and 37 were interviewed with the Kiddie Schedule for Affective Disorders and Schizophrenia (K-SADS-PL) [26]. The authors reported a prevalence rate of at least one DSM-IV disorder for 96 % of adolescents in residential units [19]. However, the proportion of interviewed adolescents living in RYC was small in this study, and interviewing only high scorers could result in biased prevalence rates. Using the same problematic two-phase design, a second British study [22] included 48 7–17 years olds looked after by a local authority and interviewed 22 CBCL high scorers with the K-SADS-PL [26].The authors reported that 21 (44 % of the total sample) had a definite, probable or resolving DSM-IV diagnosis. Given the small sample size and the methodological limitations, it is difficult to use these results in comparisons. Bronsard and colleagues (2011) assessed 183 adolescents aged 13–17 years in RYC in one county of France using the Diagnostic Interview Schedule for Children (DISC 2.25) [27] and reported that 48.6 % had fulfilled a DSM-III-R diagnosis in the previous 6 months. However, this study had a low response rate (28 %), making generalizations difficult [20]. In a Southern Sweden study, 63 % of 100 youths in four institutions received at least one clinical psychiatric diagnosis based on a systematic protocol of the investigational proceedings normally followed at the institutions, but no standardized structured psychiatric interviews were used [23]. Further, 92 % were boys, 22 of whom had been placed in coercive care according to the “Young Offenders Act”, thereby limiting the generalizability of the sample.

Schmid and colleagues included 20 of 27 institutions in the eastern part of the German state of Baden-Württemberg [21]. Half of all 1227 officially registered residential care children and adolescents aged 4–19 years were included in the study, which applied a two-step design. After screening with the CBCL/Youth Self-Report [25], 359 high scorers were interviewed using the Diagnostic System for Mental Disorders for Children and Adolescents [28]. The authors reported that 60 % of the children and adolescents fulfilled a clinical psychiatric diagnosis. However, again it is unclear if the applied screening procedure with rating scales in step one would result in reliable diagnostic prevalence estimates in the second step of the study.

To our best knowledge, there are only two studies avoiding methodological limitations in such a degree that we wanted to use them in a comparative way. The first by Keller and colleagues (2010) used the Composite International Diagnostic Interview (CIDI) [29] in a large and representative sample (*N* = 732; response rate 95 %) of youths, aged 17 years or older in child welfare agencies in three US states, 132 (18.1 %) of the youths in the sample lived in RYC. For this group, prevalence rates of 19.1 % for post-traumatic stress disorder (PTSD), 14.4 % for major depression and 10.0 % for any substance abused were estimated. Further, Ford and colleagues (2007) assessed 1543 children and adolescents aged 5–17 years who were looked after by local authorities in the British child welfare system using the DAWBA, including 279 living in RYC. They found that children and adolescents in residential care had the highest prevalence rate of any mental disorders (71 %) compared with those in foster care and other placements in the child welfare system [24].

As shown above, previous studies of residential child and youth care reported prevalence estimates of between 44 and 96 % for at least one psychiatric disorder. However, the dearth of research on reliable prevalence estimates of mental disorders in RYC gives rise to concern. Most of these studies have methodological weaknesses and lack the use of standardized psychiatric interviews conducted by trained interviewers directly in a one-to-one relationship. The present study assessed the mental health of adolescents in RYC with a structured psychiatric interview, the Child and Adolescent Psychiatric Assessment (CAPA), providing prevalence estimates for the previous 3 months according to the DSM-IV in a large representative national sample.

It is well known that prevalence rates of psychiatric disorders vary by age and sex [30–33]. Prevalence rates in the present study will therefore be reported relative to age and sex, which are not available in some of the above-cited studies of RYC. Adolescents are placed in RYC units for different juridical reasons (i.e. voluntary vs. involuntary). No study of adolescents living in RYC has so far investigated possible differences in prevalence of mental health disorders related to placement. Involuntary placement is assumed to be connected with higher rates of behavioural or drug-related problems. Research on other high-risk populations displaying conduct or drug-related problems, for example, juvenile detained offenders, has shown high comorbidity with other mental health disorders [34–36]. We therefore expect higher prevalence rates of mental disorders in adolescents who were involuntarily placed in RYC units according to the Norwegian Act of Child Protection than in those who were voluntarily placed.

Obtaining a high response rate and generalizable prevalence estimates from an adolescent population in RYC is challenging. Lower response rates are reported to be associated with lower problem levels, most likely because children with more mental health problems tend to decline or drop out of research [37]. We, therefore, for the first time, provided prevalence estimates in which adjustment is made for this non-consent bias.

The overall aim of the study was to assess the prevalence and comorbidity of mental disorders in an entire national population of adolescents living in RYC. Specific research questions were: How many residents fulfilled a psychiatric DSM-IV diagnosis during the last 3 months? What is the distribution of disorders and comorbid conditions? How are age, sex and voluntary versus involuntary placement associated with the disorder prevalence?

## Methods

### Participants

**Fig. 1:**
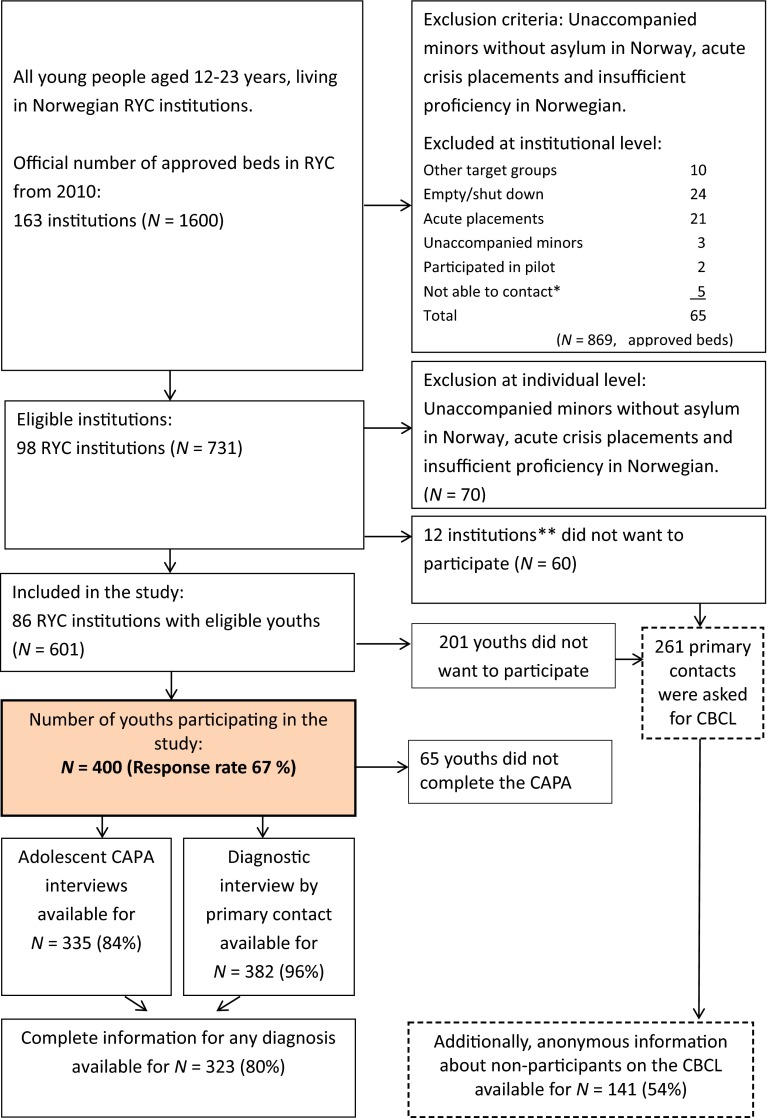
Inclusion flowchart. *CAPA* Child and Adolescent Assessment Interview, *CBCL* Child Behaviour Checklist, *primary contact* child’s individual primary contact at the institution. *The category “not able to contact” was used if institutional staff did not respond to repeated approaches about participation over a period of several months. **There were no significant differences between participating and non-participating RYC institutions with regard to geography and ownership

All residents between the ages of 12 and 23 years in RYC in Norway were invited to participate in the study (see Fig. 1). Unaccompanied minors without asylum in Norway and youths on acute placement were considered to be in such a high state of crisis that data collection should not be prioritized and were therefore excluded from the study. Youths with insufficient proficiency in Norwegian to be interviewed were also excluded. Eighty-six RYC institutions with 601 eligible youths were included. For 201 of these, the parents or youths did not consent to participate in the study, giving a total sample of 400 youths and response rate of 67 % (see Fig. 1). Table 1 shows the characteristics of the sample, consisting of 230 girls (mean age = 16.9; SD = 1.2) and 170 boys (mean age = 16.5; SD = 1.5). Of the 86 participating institutions, only 18 % had routines for regular visits from health-care workers at the institution. Regarding help for mental health problems, 86.5 % of the youths reported having ever received help from mental health services, while 37.8 % reported having received help within the last 3 months.

**Table 1:** Sample characteristics of the adolescents participating in the study

Characteristics	*n*	%	*M*	SD	Range
Gender					
Male	170				
Female	230				
Age					
Male			16.5 years	1.5 years	12.2–19.3
Female			16.9 years	1.2 years	13.5–20.2
Ethnic origin					
Norwegian	307	78.5			
1st generation immigrant	54	13.8			
2nd generation immigrant	23	5.9			
Unaccompanied minor with asylum in Norway	7	1.8			
Number of placement in the total sample	**364**		**3.34**	**2.4**	**1**–**25**
Number of placements (by decision of the child welfare system)					
1	69	19			
2	96	26.4			
3–5	150	41.2			
>5	49	13.4			
Age at first placement in the total sample	**392**		**12.5** **years**	**3.9** **years**	**0**–**17**
Age at first placement (by decision of the child welfare system)					
0–2 years	18	4.6			
3–5 years	15	3.9			
6–12 years	98	25			
13–16 years	233	59.4			
16–23 years	28	7.1			
Placement in RYC					
Voluntary	171	43.6			
Involuntary	221	56.43			
Daytime activities					
School	272	69.2			
Work	15	3.8			
Work praxis	30	7.5			
Neither school or work	70	19.5			
Parental problems					
Mother chronic illness	85	22.8			
Mother mental illness	136	36			
Mother drug use	36	9.6			
Father chronic illness	64	17.9			
Father mental illness	67	19.0			
Father drug use	43	11.8			

Total samples are indicated in bold

### Setting

RYC institutions in Norway are organized by the Norwegian Directorate for Children, Youth and Family under the Ministry of Children and Equality. The directorate is responsible for all RYC institutions,^1^ but the institutions can be both publicly and privately owned. Recently, the RYC intuitions were split into separate areas of expertise: acute, care, conduct and substance dependence. The specialized RYC institutions were created to buffer against negative and harmful influences from other residents and to tailor them to the specific challenges of the residents. Most Norwegian RYC institutions are small units with three to five residents, where the young people are encouraged to live as close to normal as possible, attending school and participating in leisure activities. The staff follow a milieu therapeutic model and have limited knowledge of psychiatric diagnosis and treatment. Child and Adolescent Mental Health Services (CAMHS) are placed under the Norwegian Directorate of Health and organized in state-owned health trusts, and a referral is needed for the screening, assessment and treatment of mental health problems.

### Procedures

The RYC institutions were contacted in randomized order by the research team. Data collection in the RYC institutions was carried out by four trained research assistants and the collection lasted approximately 4 h per youth. Due to the length of CAPA and the adolescents’ considerable challenges related to concentration and stamina, not all residents were able to complete the psychiatric interview. In addition, the child’s primary contact at the institution was interviewed by the research assistant about the child’s attention deficit hyperactivity disorder (ADHD) and Asperger symptoms (AS) and symptoms of reactive attachment disorder (RAD) (see also below). The child’s primary contact also reported each resident’s mental health problems by means of the CBCL. The Norwegian Regional Committee for Medical and Health Research Ethics also approved the collection of anonymous ratings from the primary contacts for an attrition analysis on the CBCL for residents who did not participate in the study; such anonymous information was provided for 141 adolescents (see also Fig. 1 and under “Ethics”). In addition, the heads of the institutions completed a questionnaire giving factual information about the RYC institution. The data were collected from June 2011 to July 2014.

### Instruments

#### Psychiatric disorders

The CAPA is an interviewer-based semi-structured psychiatric interview that collects data on the onset dates, duration, frequency and intensity of symptoms of a wide range of psychiatric diagnoses, according to the DSM-IV [38]. In addition, assessment of received child health services can be obtained. The interview serves as a guide in determining whether a symptom is present at prespecified levels and the interviewer is expected to probe until she or he can decide whether the symptom is present. Information concerning the frequency, onset, intensity and duration is obtained. Moreover, functional impairment is evaluated. The test–retest reliability has been shown to be adequate, ranging from *κ* = 0.55 for conduct disorders (CD) to 1 for substance abuse/dependence [38]. Training of the interviewer and coding are based on a detailed glossary, which defines each symptom and the criteria for coding different levels of symptom severity on several dimensions. Interviewers (*N* = 4) had at least a bachelor’s degree in relevant fields and extensive prior experience in working with children and families. Regular supervision with master coders was held. In the present study, more than 10 % of audiotaped youth (CAPA) or primary contact interviews (*N* = 42) were randomly selected for recoding by a randomly selected other interviewer. Interrater reliability for the rater pairs as estimated by Gwet’s AC_1_ (and agreement rate) were: AS = 0.83 (88 %); ADHD = 0.74 (83 %); CD = 0.78 (86 %); oppositional defiant disorder (ODD) = 0.97 (98 %); RAD = 0.82 (88 %); substance abuse = 0.69 (76 %); major depression disorder (MDD) = 0.89 (93 %); dysthymia = 0.92 (95 %); agoraphobia without panic = 1.0 (100 %); specific phobia = 0.86 (88 %); social phobia = 0.87 (91 %); obsessive–compulsive disorder (OCD) = 1.0 (100 %); and generalized anxiety disorder (GAD) = 0.93 (95 %). Those not reported did not occur in this random subsample, and grouped diagnoses were not analysed.

There is an ongoing academic discussion regarding whether self-reports in children with ADHD and AS are reliable or if children evaluate themselves too positively [39–41]. We considered adolescents to be poorer reporters of symptoms with regard to ADHD and AS than adults who knew them well. Information about symptoms and diagnostic criteria of ADHD and AS was therefore obtained in interviews with the youths’ primary contact at the institution using the Parent version of the CAPA interview [38]. Further, a previously given ADHD diagnosis by a specialist in child psychiatry or paediatrics was accepted, even if the CAPA interview reported only sub-threshold symptoms. ADHD symptoms could have been reduced to sub-threshold levels by current medication or other therapeutic interventions for ADHD at the time of data collection. Therefore, we also specified separately the reporting rate for “Clinical diagnoses/treatment for ADHD” and the combined information; “ADHD total”.

Because the CAPA does not cover pervasive developmental disorders, primary contacts were interviewed using the Asperger Syndrome Diagnostic Interview (ASDI) [42]. The relevant items contributed a DSM-IV-based algorithm for the AS diagnoses.

The validity and relevance of the criteria for the diagnosis of RAD have been controversial, especially after the age of 5 years [43], and RAD is one of the least researched and most poorly understood disorders in the DSM [44]. Further, the CAPA does not cover RAD. In the present study, we therefore diagnosed RAD with selected questions from the preschool age version (PAPA) [45] of the CAPA by interviewing the child’s primary contact at the institution. Three questions were considered inadequate in describing maladaptive adolescent behaviour such as ‘negative reunion response’, ‘do not seek comfort’ and ‘frozen watchfulness’ and were therefore excluded. Our sample consisted of adolescents in RYC institutions where parental information about the individual respondent’s behaviour before the age of five was not available (DSM-IV criterion A). However, most of the adolescents were placed in these institutions because of severe psychosocial strains and child maltreatment requiring the type of care beyond that which foster homes can manage. Thus, our diagnostic adaptations represent several limitations to the study. On the other hand, there exists very little systematically gathered epidemiologic information on RAD [44], and we decided to include RAD despite those limitations.

#### Symptom screening

The Child Behaviour Checklist (CBCL) consists of 118 Likert-type and two open-ended items rated on a 0–2 scale (0 = not true, 1 = somewhat or sometimes true, or 2 = very true or often true). For the present study, we used the following eight syndrome scales of the 2001 version [25] for children and adolescents aged 6–18 years: Anxious/depressed, Withdrawn/depressed, Somatic complaints, Social problems, Thought problems, Attention problems, Rule-breaking behaviour and Aggressive behaviour. The Norwegian version of the CBCL showed satisfactory reliability and validity (α of 0.93 for the Total problems scale, and 0.84 and 0.89 for the Internalizing and Externalizing subscales, respectively) [46, 47]. In the current sample α was 0.96 for the Total problems scale. For the subscales it was 0.84 for Anxious/depressed, 0.75 for Withdrawn/depressed, 0.80 for Somatic complaints, 0.77 for Social problems, 0.77 for Thought problems, 0.80 for Attention problems, 0.86 for Rule breaking and 0.92 for Aggressive behaviour.

#### General institutional information

Information about the RYC institutions was obtained by a questionnaire answered by the institutional leaders.

### Statistics

The CBCL available for both participating and 141 non-participating anonymous youths (only age and sex available) was used for an adjustment of observed prevalence because of missing diagnostic categories and attrition due to non-consent (see Fig. 1). We substituted missing values on all DSM-IV diagnoses with Bayesian multiple imputation (MI), which is the recommended approach with categorical data that are not missing completely at random [48]. To increase precision, all missing values on diagnostic categories were substituted by MI with 100 imputed data sets [49] and were based on variables in the data set assumed to be relevant predictors for missing values, such as sex, age and the eight CBCL syndrome scales of the CBCL available for both participants and non-participants. Thus, it was possible to estimate complete DSM-IV diagnoses for 541 adolescents. Comparing these prevalence rates with the observed prevalence informed us about the consequences of missing data and attrition on prevalence in our study. MI was conducted with Mplus version 7.2 (Muthén and Muthén, 1998–2014). There were significantly (*p* = 0.001) more girls (*n* = 230) among participants than boys (*n* = 170) compared to non-participants (56 girls versus 81 boys, *n* = 4 missing information on sex on CBCL form), while we did not find any significant differences with regard to age. We also compared participants with non-participants on the eight CBCL subscales showing that non-participants had significantly higher scores on five of eight subscales (see Table 2). However, Pearson’s effect sizes (*r*) for these differences were all small, as interpreted by Cohen where *r* < 0.30 represents small effects, *r* = 0.30–0.50 moderate effects and *r* > 0.50 large effects [50]. These differences representing possible attrition bias have been corrected in the MI, since we used all CBCL information (from both participants and non-participants) as auxiliary variables for the prediction of missing CAPA diagnoses both for participants and non-participants.

**Table 2:** Comparison of participants versus non-participants on eight CBCL subscales

	Anxious/depressed	Withdrawn/depressed	Somatic complaints	Social problems	Thought problems	Attention problems	Rule breaking	Aggressive behaviour
	Mean (SD)	Mean (SD)	Mean (SD)	Mean (SD)	Mean (SD)	Mean (SD)	Mean (SD)	Mean (SD)
Participants (*n* = 353–359)	6.6 (4.9)	4.7 (3.2)	4.1 (3.8)	4.6 (3.7)	4.4 (3.8)	7.3 (4.2)	9.4 (6.0)	10.3 (7.8)
Nonparticipants (*n* = 140–141)	7.2 (5.1)	5.6 (3.1)	3.8 (3.8)	5.3 (3.8)	5.4 (4.6)	8.6 (4.1)	10.8 (7.0)	12.2 (8.1)
*p* value	0.218	**0.007**	0.516	0.079	**0.021**	**0.003**	**0.041**	**0.016**
Effect size (*r*)*	0.05	0.12	0.02	0.07	0.11	0.13	0.09	0.11
Range of subscale	0–26	0–16	0–22	0–22	0–30	0–20	0–34	0–36

OR in bold = *p* < 0.05

* *r* = 0.10–0.30 = small effect

Rater agreement was evaluated by calculating Gwet’s AC_1_. Some diagnoses in the present study were low prevalent. Gwet’s AC1 was chosen because Cohen’s kappa has the paradoxical property of giving low values when the prevalence is low, even if the raters agree highly. Gwet’s AC1, on the other hand, adjusts for agreement by chance in a way that resolves this paradox [51]. Gwet’s AC_1_ was calculated in AggreeStat (supplied commercially by Gwet at http://www.agreestat.com/agreestat.html).

To investigate how age, sex and voluntary versus involuntary placement were associated with the prevalence of disorders, logistic regression analysis was used. A significance level of *p* < 0.05 was chosen. All other statistical analyses were conducted with SPSS version 21.

### Ethics

Participants were recruited using procedures approved by the Norwegian Regional Committee for Medical and Health Research Ethics and written consent was always obtained. If the young person was under 16 years, informed consent from the significant caregiver was also acquired. The Norwegian Regional Committee for Medical and Health Research Ethics had also approved the collection of ratings on the CBCL of participants and non-participants from the child’s primary contact at the institution provided, regardless of whether or not the resident gave their consent to participate in the study. For anonymous non-participants, only age, sex and CBCL items were available. After the primary contact at a given institution had completed the CBCL for a non-participating adolescent, it was sent without any identifying information in a standard envelope to a secretary who was not a member of the research group for collection and locked in storage until all data collection in the project was completed.

## Results

### Prevalence of any DSM-IV psychiatric disorder

We observed 76.2 % of youths living in RYC in Norway fulfilling the symptom, onset, duration and impairment criteria for at least one DSM-IV diagnosis during the previous 3 months (see Table 3) calculated from complete information cases. This observed prevalence rate was confirmed by MI estimation among 400 participants combined with CBCL information of 141 non-participants, showing a prevalence rate of 76.0 % (*N* = 541). The estimated prevalence rates for *N* = 541 are reported in detail in Table 3, showing only small deviance from observed prevalence, well within its 95 % confidence limits for all main diagnoses and larger diagnostic categories. Excluding RAD from the overall observed prevalence for at least one DSM-IV diagnosis only reduced it from 76.2 to 74.5 %.

**Table 3:** Observed 3-month prevalence of psychiatric DSM-IV disorders in percentages (95 % confidence interval) according to gender, age and voluntary vs. involuntary placement status

	*N*	Observed^b^ prevalence % (*N* = 323–399)	Estimated^c^ prevalence % (*N* = 541)	Gender^a^			Age^a^			Placement status^a^		
				Boys % (Cl 95 %)	Girls % (Cl 95 %)	OR, *p* value	12–16 % (Cl 95 %)	17–21 % (Cl 95 %)	OR, *p* value	Voluntary % (Cl 95 %)	Involuntary % (Cl 95 %)	OR, *p* value
AS	323	23.2 (18.9–28.1)	24.4	26.3 (19.6–34.4)	21.1 (15.8–27.4)	1.3, *p* = 0.271	22.5 (16.7–29.6)	23.9 (18.0–31.0)	1.1, *p* = 0.761	19.4 (13.7–26.7)	26.7 (20.7–33.7)	1.5, *p* = 0.132
ADHD (CAPA)	382	13.4 (10.3–17.1)	–	15.5 (10.7–21.9)	11.8 (8.2–16.7)	1.4, *p* = 0.287	13.7 (9.6–19.2)	13.0 (8.9–18.6)	0.9, *p* = 0.833	12.8 (8.5–18.8)	13.3 (9.4–18.6)	1.1, *p* = 0.881
Clinical ADHD/treatment	399	24.8 (20.8–29.3)	–	27.6 (21.5–34.8)	22.7 (17.7–28.6)	1.3, *p* = 0.259	26.1 (20.6–32.5)	23.4 (18.0–29.9)	0.9, *p* = 0.541	25.1 (19.2–32.1)	24.5 (19.3–30.6)	1.0, *p* = 0.891
ADHD total	399	32.3 (27.9–37.1)	33.1	37.1 (30.2–44.5)	28.8 (23.3–35.0)	1.4, *p* = 0.083	31.4 (25.5–38.0)	33.3 (27.0–40.3)	1.1, *p* = 0.680	32.2 (25.6–39.5)	32.7 (26.9–39.2)	1.0, *p* = 0.906
CD	335	19.1 (15.2–23.6)	17.9	28.8 (21.9–36.8)	12.2 (8.4–17.6)	**2.9,** ***p*** **=** **0.001**	21.7 (16.1–28.5)	16.6 (11.7–22.9)	0.7, *p* = 0.235	9.7 (5.8–15.5)	26.4 (20.5–33.2)	**3.4,** ***p*** **=** **0.001**
ODD	335	3.0 (1.6–5.4)	2.2	1.4 (0.3–5.1)	4.1 (2.1–7.8)	0.3, *p* = 0.180	3.6 (1.7–7.7)	2.4 (0.9–5.9)	0.6, *p* = 0.505	2.8 (1.1–6.9)	3.3 (1.5–7.0)	1.2, *p* = 0.779
RAD	323	21.1 (17.6–25.8)	23.7	18.0 (12.4–25.4)	23.2 (17.7–29.7)	0.7, *p* = 0.268	23.8 (17.8–30.9)	18.4 (13.2–25.1)	0.7, *p* = 0.240	16.5 (11.3–23.6)	25.6 (19.7–32.5)	1.7, *p* = 0.055
Substance abuse	335	11.9 (8.9–15.9)	12.0	12.2 (7.7–18.7)	11.7 (7.9–17.0)	1.0, *p* = 0.890	12.7 (8.4–18.6)	11.2 (7.3–16.9)	0.9, *p* = 0.691	9.7 (5.8–15.6)	14.3 (9.9–20.1)	1.6, *p* = 0.207
Substance dependence	335	2.7 (1.4–5.0)	2.6	0.7 (0.1–4.0)	4.1 (2.1–7.8)	0.2, *p* = 0.097	2.4 (0.9–6.0)	3.0 (1.3–6.7)	1.2, *p* = 0.756	2.8 (1.1–6.9)	2.7 (1.2–6.3)	1.0, *p* = 0.995
MDD	335	23.3 (19.1–28.1)	21.6	10.8 (6.6–17.0)	32.1 (26.0–39.0)	**0.3** ***p*** **=** **0.001**	24.7 (18.8–31.8)	21.9 (16.3–28.7)	0.9, *p* = 0.544	20.7 (14.9–28.0)	25.3 (19.5–32.1)	1.3*, p* = 0.330
Dysthymia^d^	335	30.1 (25.5–35.3)	29.0	14.4 (9.5–21.2)	41.3 (34.6–48.3)	**0.2,** ***p*** **=** **0.001**	31.3 (24.7–38.7)	29.0 (22.7–36.2)	0.9, *p* = 0.642	24.8 (18.5–32.4)	33.5 (27.1–40.7)	1.5, *p* = 0.088
Depression NOS	335	24.8 (20.5–29.7)	22.7	16.5 (11.3–23.6)	30.6 (24.6–37.3)	**0.5,** ***p*** **=** **0.004**	28.3 (22.0–35.6)	21.3 (15.8–28.1)	0.7, *p* = 0.138	20.0 (14.3–27.2)	27.5 (21.5–34.4)	1.5, *p* = 0.118
Bipolar disorder	334	0.6 (0.1–2.0)	0.6	0.0 (0–0)	1.0 (0.3–3.7)	0.0, *p* = 0.996	0.0 (0–0)	1.2 (0.3–4.2)	0.0, *p* = 0.996	0.7 (0.1–3.8)	0.5 (0.1–3.0)	0.8, *p* = 0.868
MDD, dep NOS or dysthymia^e^	335	37.0 (32.0–42.3)	–	22.3 (16.2–29.3)	47.4 (40.6–54.4)	**0.3,** ***p*** **=** **0.001**	39.2 (32.1–46.7)	34.9 (28.1–42.4)	0.8, *p* = 0.413	30.3 (23.4–38.3)	41.8 (34.8–49.0)	1.6, *p* = 0.059
AG without panic	333	12.6 (9.5–16.6)	12.9	8.7 (5.0–14.6)	15.4 (11.0–21.1)	0.5, *p* = 0.074	10.4 (6.6–16.0)	14.8 (10.2–20.9)	1.5, *p* = 0.226	11.0 (6.9–17.2)	13.9 (9.6–19.7)	1.3, *p* = 0.442
Panic without AG	333	3.9 (3.3.–6.6)	3.5	0.0 (0.0–0.0)	6.7 (3.9–11.1)	0.0, *p* = 0.996	4.9 (2.5–9.3)	3.0 (1.3–6.7)	0.6, *p* = 0.371	2.8 (1.1–6.9)	5.0 (2.7–9.2)	1.9, *p* = 0.312
Panic with AG	333	0.9 (0.3–2.6)	0.9	0.0 (0.0–0.0)	1.5 (0.5–4.4)	0.0, *p* = 0.996	0.6 (0.1–3.4)	1.2 (10.2–20.9)	1.9, *p* = 0.586	0.7 (0.1–3.8)	1.1 (0.3–4.0)	1.6, *p* = 0.696
Specific phobia	335	6.3 (4.1–9.4)	5.9	3.6 (1.5–8.1)	8.2 (5.0–12.8)	0.4, *p* = 0.098	5.4 (2.9–10.0)	7.1 (4.1–12.0)	1.3, *p* = 0.527	6.2 (3.3–11.4)	6.0 (3.4–10.5)	1.0, *p* = 0.951
Social phobia	335	12.5 (9.4–16.5)	13.7	7.2 (4.0–12.7)	16.3 (11.8–22.1)	**0.4,** ***p*** **=** **0.015**	12.0 (7.9–17.9)	13.0 (8.8–18.9)	1.1, *p* = 0.789	12.4 (8.0–18.8)	12.6 (8.6–18.2)	1.0, *p* = 0.952
OCD	335	3.6 (2.1–6.2)	3.1	2.2 (0.7–6.2)	4.6 (2.4–8.4)	0.5, *p* = 0.249	2.4 (0.9–6.0)	4.7 (2.4–9.1)	2.0, *p* = 0.261	4.1 (2.9–8.7)	3.3 (1.5–7.0)	0.8, *p* = 0.688
PTSD	335	0.6 (0.2–2.2)	0.4	0.7 (0.1–3.9)	0.5 (0.09–2.8)	1.4, *p* = 0.807	0.6 (0.1–3.3)	0.6 (0.1–3.3)	1.0, *p* = 0.990	0.7 (0.1–3.8)	0.5 (0.1–3.0)	0.8, *p* = 0.872
GAD	335	20.9 (16.9–25.6)	20.7	15.1 (10.0–21.9)	25.0 (19.5–31.5)	**0.5,** ***p*** **=** **0.030**	21.1 (15.6–27.9)	20.7 (15.3–27.4)	1.0, *p* = 0.933	15.9 (10.8–22.7)	25.3 (19.5–32.1)	**1.8,** ***p*** **=** **0.040**
Bulimia	335	0.9 (0.3–2.5)	0.7	0.0 (0–0)	1.5 (0.5–4.4)	0.0, *p* = 0.996	1.2 (0.3–4.3)	0.6 (0.1–3.3)	0.5, *p* = 0.560	1.4 (0.4–4.9)	0.5 (0.1–3.0)	0.4, *p* = 0.450
Any anxiety disorder	335	34.0 (29.2–39.2)	–	25.9 (19.3–33.7)	39.8 (33.2–46.8)	**0.6,** ***p*** **=** **0.028**	31.3 (24.7–38.7)	36.7 (29.8–44.2)	1.2, *p* = 0.491	26.2 (19.7–33.9)	40.1 (33.3–47.4)	**1.9,** ***p*** **=** **0.014**
Any disorder	**323**	**76.2** **(71.2–80.1)**	**76.0**	73.7 (65.6–80.0)	77.9 (71.5–83.2)	0.8, *p* = 0.383	76.9 (69.7–82.7)	75.5 (68.3–81.4)	0.9, *p* = 0.765	68.3 (60.2–75.4)	81.8 (75.4–86.8)	**2.1,** ***p*** **=** **0.006**

OR in bold = *p* < 0.05

Estimated prevalence of missing cases inclusive of non-participants (*N* = 541)

*AS* Asperger’s syndrome, *ADHD (CAPA)* attention deficit disorder as obtained by CAPA interview, clinical ADHD/treatment, diagnosis set before the current study by a specialist in child psychiatry or paediatrics and/or presently medicated for ADHD, *ODD* oppositional defiant disorder without CD exclusion, *CD* conduct disorder, *RAD* reactive attachment disorder, *MDD* major depressive disorder, *dep NOS* depressive disorder not otherwise specified, *GAD* generalized anxiety disorder, panic with *AG* agoraphobia, *OCD* obsessive compulsive disorder, *PTSD* post-traumatic stress disorder; substance abuse and dependency do not include tobacco

^a^Calculations for age, gender and placement status were based on observed prevalence. Parameter coding for girls = 0, boys = 1; 12–16 years = 0, 17–21 years = 1; voluntary = 0, involuntary = 1

^b^Observed prevalence was calculated based on complete case information of *N* = 323–399 participants

^c^Estimation by multiple imputation (MI) for *N* = 541 was based on maximum available information for each participant and 141 non-participants. (CIs not available with MI in Mplus)

^d^Dysthymia prevalence rate was calculated without duration criteria

^e^Dysthymia prevalence rate was calculated according to DSM-IV 1 year duration criteria

### Distribution of DSM-IV disorders and comorbid conditions

According to Table 3, the most frequent diagnoses or diagnostic categories observed were MDD or depression NOS or dysthymia (37.0 %), followed by any anxiety disorder (34.0 %), ADHD (32.3 %) and AS (23.2 %). We observed a prevalence rate for CD of 19.1 %. RAD was diagnosed in 21.1 % of the youths, and we estimated the prevalence to be as high as 23.7 %. Surprisingly, only 0.6 % fulfilled a PTSD diagnosis, in spite of an observed high rate of reported potentially traumatic events (79 %) and for many this was combined with avoidance related to these events. We did not observe any diagnoses of Tourette syndrome or anorexia nervosa.

Table 4 shows the prevalence and odds ratios for a range of comorbid common diagnostic categories. For instance, the first filled cell in Table 4 (line 1, column 2) shows that 59.8 % of those with any affective disorder had a comorbid anxiety disorder and that the odds of having any anxiety disorder were 5.6 times higher in those with any affective disorder than in those without. Comorbidity in our study was high. Of those with any disruptive behavioural disorder (CD, ODD), 52.2 % also had any affective disorder and 47.8 % had any anxiety disorder. Over half of those with any anxiety disorder also had an affective disorder and vice versa. Half of those with substance disorders also had any affective, anxiety or disruptive behaviour disorder (see Table 4).

**Table 4:** Comorbidity between common diagnostic categories. Prevalence (percent) and odds ratio (OR) (95 CI), *N* = 323

	Any affective disorder	Any anxiety disorder	ADHD	Any disruptive behaviour disorder	Any substance disorder
Any affective disorder	**–**	59.8 % **5.6 (3.4**–**9.3)**	35.2 % 1.3 (0.8–2.1)	29.5 % **2.1 (1.2–3.7)**	17.2 % 1.8 (0.9–3.4)
Any anxiety disorder	63.5 % **5.6 (3.4**–**9.3)**	**–**	33.0 % 1.1(0.7–1.8)	28.7 % **1.9 (1.1–3.3)**	18.3 % **2.0 (1.0–3.8)**
ADHD	42.2 % 1.3 (0.8–2.1)	37.3 % 1.1 (0.7–1.8)	**–**	29.4 % **1.9 (1.1–3.4)**	15.7 % 1.4 (0.7–2.7)
Any disruptive behaviour disorder	52.2 % **2.1 (1.2**–**3.7)**	47.8 % **1.9(1.1–3.3)**	43.5 % **1.9 (1.1–3.4)**	**–**	31.9 % **5.5 (2.8–10.8)**
Any substance disorder	50.0 % 1.8 (0.9–3.4)	50.0 % **2.0 (1.0–3.8)**	38.1 % 1.4 (0.7–2.7)	52.5 % **5.5 (2.8–10.8)**	**–**

OR in bold = *p* < 0.05

### Sex, age and voluntary versus involuntary placement

Table 3 displays differences in prevalence for sex, in that girls had lower odds of receiving a DSM-IV CD diagnosis than boys, but had over three times higher odds of suffering depression or dysthymia, with odds ratio (OR) = 0.3 for boys versus girls. Girls also had 2.5 times higher odds of having social phobia and five times higher odds of suffering from dysthymia than boys (see Table 2, OR = 0.4 and 0.2, respectively). We did not observe any significant age differences with regard to DSM-IV diagnoses. With regard to placement status, youths who were placed involuntarily had over three times higher odds of receiving a CD diagnosis than voluntarily placed residents (Table 3). They also had almost two times higher odds of suffering from at least one anxiety disorder and of receiving any DSM-IV diagnosis.

## Discussion

In this nationwide study, we found the exceptionally high prevalence rate of 76 % with at least one DSM- IV diagnosis during the 3 months prior to the interview. Comorbidity was common. Overall, we observed higher prevalence rates for depressive and anxiety psychiatric disorders than for behavioural disorders, but also high comorbidity between depressive/anxiety and behavioural disorders. Being placed involuntarily was associated with three times higher odds of having a CD, and two times higher odds of having an anxiety disorder and for having a DSM-IV diagnosis.

### Overall prevalence of disorder

Compared with the two studies of psychiatric disorders in youths in RYC with least methodological limitations relevant for such comparison, our overall prevalence rate of 76 % for any psychiatric disorder is close to the findings of the British study (71 %) by Ford et al. (2007). The Keller et al. study did not report an overall prevalence of any DSM-IV diagnosis. There are some limitations of the comparison of prevalence rates of mental health disorders due to differences in culture, legislation, institutional capacities and organization of mental health and child welfare systems in different countries. In addition, our study is the only one reporting prevalence on RAD in RYC. However, without RAD our overall prevalence rate is 74.5 %, still somewhat higher than in the British study.

Norwegian prevalence estimates for adolescents and young adults in the general population are unfortunately not available using a similar methodological approach as ours. A randomly selected control group of 75 20-year-olds in a Norwegian study of prematurely born youths [52] was assessed with the Schedule for Affective Disorders and Schizophrenia for School-age children, Present and Lifetime version (K-SADS-PL) [26] and reported an 8 % prevalence rate of overall psychiatric disorders. A large Norwegian study using the PAPA in preschoolers showed an estimated population rate for any psychiatric disorder of 7.1 % [53]. Thus, our 3-month prevalence rate of overall psychiatric disorders was about ten times higher than in these two studies.

### Prevalence of specific disorders

Our prevalence for MDD of 23.3 % was higher than the 10.5 % found by Keller et al. (2010). The British study by Ford et al. (2007) reported that 18.6 % had an emotional disorder in RYC, whereas our results showed the much higher prevalence rates of 37.0 % suffering from depression or dysthymia and 34.0 % of any anxiety disorder among the residents. In contrast to the British study showing 10 % ADHD, we observed higher overall prevalence rates for ADHD (32.3 %), including higher rates of ADHD diagnoses set by clinicians before assessment with the CAPA (24.8 %) as well as remaining diagnosable ADHD disorders (13.4 %) among residents. On the contrary, the British study found 61.3 % of residents having CD, while our prevalence rates were only one-third of this rate (19.1 %). So even if the overall prevalence for any psychiatric diagnose was similar in our study to the British, the distribution of specific disorders was different in these two studies.

In contrast to the above-mentioned control group of young adults [52] reporting 3 % anxiety and 3 % mood disorders, our prevalence figures were 11–12 times higher than those found in that community sample.

Keller et al. (2010) reported lifetime prevalence for PTSD of 15.1 % in RYC. Given the high observed prevalence of potentially traumatic events in our study, we were surprised that only two (0.6 %) of the adolescents fulfilled a PTSD diagnosis according to the DSM-IV. The high frequency of avoidance observed in combination with traumatic exposure may indicate that these strains have resulted in other conditions than PTSD, such as depression and/or anxiety. Investigating this issue further, we consider being outside the scope of this paper.

We have not found any reports of RAD in previous studies of adolescents in RYC. This diagnosis is assessed mostly in pre-schoolers and is therefore not included in any of the commonly used structural psychiatric interviews for adolescents, such as the CAPA. However, we expected that youths in the child welfare system would have experienced severe psychosocial strains and child maltreatment resulting in RAD, which would have persisted into adolescence and young adulthood. Our modified version of the PAPA items yielded an observed prevalence rate of 21.1 % for RAD. A recent Norwegian study of 6- to 12-year-old foster children reported an RAD prevalence of 19.4 % [43]. Our age adjustments of the DSM-IV criteria have not been empirically validated, so conclusions regarding this diagnosis should be made with caution. However, our results emphasize that essential features of the RAD diagnosis do not disappear after preschool age, but persist into adolescence and young adulthood. Further research is called for to improve the validity and reliability of the RAD diagnosis and possibly extending its use beyond childhood. This would be important given the lack of research and the ensuing critique with regard to this diagnosis. It is one of the most poorly understood disorders in the DSM [44], but still a potentially important perspective for understanding the development and problems of those growing up under neglect and maltreatment.

It is not surprising that depression and anxiety showed very high comorbidity, because this relationship has been well established in child and adolescent psychiatry [54]. However, the observed high comorbidity between behavioural and anxiety/emotional disorders in our study gives serious cause for concern for the design of RYC institutions and will be discussed further.

### Sex, age and placement status

As expected, our findings showed depression and anxiety to have a marked female preponderance, in accordance with established knowledge [30–33], and males had higher odds of receiving a CD diagnosis. Surprisingly, we did not find any age differences with regard to mental disorders, although increasing age trends exist for many psychiatric diagnoses through adolescence in community populations. The observed differences related to voluntary/involuntary placement reflect a placement practice resulting in involuntary placement of adolescents with CD. Our results also point out that adolescents placed involuntarily often also suffer from anxiety that may be overlooked due to the more visible conduct problems. This represents important knowledge for those working in RYC.

### Consequences of designing residential child welfare institutions

Our study revealed that three-fourths of youths living in RYC actually suffered a psychiatric disorder with impairment of functioning during the previous 3 months while they were in a child welfare residential institution, and were not placed in a mental health institution. Only 18 % of the institutions had routines for regular visits from health-care workers to the institution and only 37.8 % of the residents had actually received help from mental health services during the last 3 months. In RYCs, the staff are mainly educated and competent in providing a positive social environment, physical custody, care and control, but not psychiatric diagnostic assessment and therapy. Thus, it is essential to secure systematic psychiatric assessment for these residents by institutional routines. In addition, systematic organizational cooperation between RYCs and youth mental health services is required, but is rare in the Norwegian system. We agree with Bronsard and colleagues (2011) that the integration of systematic mental health screening is required for youths entering the child welfare system [20], especially before placement in RYC. It is critically important to assess the level and type of mental health needs of children and youths across all sectors to deliver the most effective combined services [55].

Further, we found considerable comorbidity between anxiety, depressive disorders and serious behavioural disorders, reflecting the complexity of the difficulties these youths face. The fact that effective evidence-based psychiatric interventions for adolescents are available for each of the psychiatric disorders leads to an ethical and health political dilemma, since the existing method of organizing RYC seems to limit the availability of psychiatric services. Given the existing limitations to psychiatric competency among the staff in RYC institutions and the observed high comorbidity between internalizing and externalizing disorders, it is a major challenge to implement individually tailored psychiatric therapeutic interventions and mileu therapy. The observed prevalence of attachment disorder is close to five times the maximum 5 % observed in usual adolescent mental health services (unpublished study of the Health Survey in the Department of Child and Adolescent Psychiatry, St. Olav’s University Hospital, Norway). Adolescents with attachment problems have a need for continuity of relationships over longer periods, to develop trust and confidence in predictable adult care persons. These are needs that can seldom be met in CAHMS, which is a part of an effective modern specialized health-care system dominated by therapeutic short-time interventions.

Specially designed institutions for youths with serious behavioural problems have been established recently to improve RYC quality. However, the observed high comorbidity in our study challenges the idea of selecting and designing institutions for one dominating disorder. Designing alternative RYC institutions especially for internalizing disorders would also be a bad idea, given the observed comorbidity between affective/anxiety disorders and disruptive/substance disorders.

In a social–economic cost–benefit perspective, practitioners and policy makers should have a significant interest in the mental health status of adolescents in RYC units, because major mental health and substance use disorders in youths contribute to impairments in functioning during early adulthood [56]. Therefore, a thorough evaluation and reorganization of the RYC system based on reliable prevalence rates of mental disorders is called for. Further studies should also investigate what kind of help youths in RYC actually receive and the adequacy of this help in regard to their mental health status.

## Limitations

We did not have the opportunity to include the adolescents’ parents as informants, thereby limiting our knowledge about early development and family functioning before placement in RYC. Thus, the requirement in some diagnoses as RAD for developmental continuity and early onset could not be used. Further, to obtain information about ADHD, we had to rely on each adolescent’s primary contact at the institution for CAPA diagnostic criteria, combined with teacher reports and the adolescent’s self-report with regard to previous ADHD diagnoses and medication prescriptions. Including the assessment of AS and RAD required modifications of original instruments, because these categories were not covered by the CAPA interview, which also represents a limitation of the study. Another limitation was that primary contacts and teachers were not used as additional informants on the adolescents’ CD and ODD symptoms, as a supplement to the interview of the residents. This procedure may have led to an underreporting of ODD and CD in the current sample. We found that non-participants had slightly higher problems on five of eight CBCL subscales and girls were overrepresented among participants, thereby introducing bias regarding the observed prevalence figures. However, since we used this CBCL information of both participants and non-participants to predict missing CAPA diagnoses by the MI method, these biases were to a large degree corrected in the reported estimated prevalence.

## Conclusion

This study directly assessed the mental health of adolescents in RYC by trained professionals with a standardized psychiatric interview. The high prevalence of mental disorder among adolescents in RYC (76.2 %) causes major concerns and challenges the existing organization of the RYC system. The observed high comorbidity in our study contradicts the idea of selecting and designing institutions for one dominating disorder. A systematic mental health screening should be carried out before youths are placed in RYC institutions. In addition, systematic psychiatric assessment and treatment for residents should be an integrated part of institutional routines. Further research is needed to identify effective interventions addressing the complexity of these youths’ mental health problems.

## Footnotes

1 Except for RYC in the municipality of Oslo, which administers its own RYC institutions.
